# Canakinumab relieves symptoms of acute flares and improves health-related quality of life in patients with difficult-to-treat Gouty Arthritis by suppressing inflammation: results of a randomized, dose-ranging study

**DOI:** 10.1186/ar3297

**Published:** 2011-03-25

**Authors:** Naomi Schlesinger, Marc De Meulemeester, Andrey Pikhlak, A Eftal Yücel, Dominik Richard, Valda Murphy, Udayasankar Arulmani, Peter Sallstig, Alexander So

**Affiliations:** 1 Division of Rheumatology, Department of Medicine, Robert Wood Johnson Medical School, 125 Patterson Street, New Brunswick, NJ 089010, USA; 2 Pratique Médicale, Cabinet de Là-Haut, Rue de Marchienne 113, 6534 Gozée, Belgium; 3 Moscow State University of Medicine and Dentistry, Clinical-Diagnostic Center MSMSU, Dolgorukovskaya Street 4, Moscow 127006, Russia; 4 Baskent University, Faculty of Medicine, Baglica Kampusu, Eskisehir Yolu 20.km, Baglica 06530, Ankara, Turkey; 5 Immunology & Infectious Disease Therapeutic Area, Novartis Pharma AG, Lichtstrasse 35, CH-4056, Basel, Switzerland; 6 Service de Rhumatologie, Centre Hospitalier Universitaire Vaudois, University of Lausanne, Av. Pierre-Decker 5, CH-1005, Lausanne, Switzerland

## Abstract

**Introduction:**

We report the impact of canakinumab, a fully human anti-interleukin-1β monoclonal antibody, on inflammation and health-related quality of life (HRQoL) in patients with difficult-to-treat Gouty Arthritis.

**Methods:**

In this eight-week, single-blind, double-dummy, dose-ranging study, patients with acute Gouty Arthritis flares who were unresponsive or intolerant to - or had contraindications for - non-steroidal anti-inflammatory drugs and/or colchicine were randomized to receive a single subcutaneous dose of canakinumab (10, 25, 50, 90, or 150 mg) (*N *= 143) or an intramuscular dose of triamcinolone acetonide 40 mg (*N *= 57). Patients assessed pain using a Likert scale, physicians assessed clinical signs of joint inflammation, and HRQoL was measured using the 36-item Short-Form Health Survey (SF-36) (acute version).

**Results:**

At baseline, 98% of patients were suffering from moderate-to-extreme pain. The percentage of patients with no or mild pain was numerically greater in most canakinumab groups compared with triamcinolone acetonide from 24 to 72 hours post-dose; the difference was statistically significant for canakinumab 150 mg at these time points (*P *< 0.05). Treatment with canakinumab 150 mg was associated with statistically significant lower Likert scores for tenderness (odds ratio (OR), 3.2; 95% confidence interval (CI), 1.27 to 7.89; *P *= 0.014) and swelling (OR, 2.7; 95% CI, 1.09 to 6.50, *P *= 0.032) at 72 hours compared with triamcinolone acetonide. Median C-reactive protein and serum amyloid A levels were normalized by seven days post-dose in most canakinumab groups, but remained elevated in the triamcinolone acetonide group. Improvements in physical health were observed at seven days post-dose in all treatment groups; increases in scores were highest for canakinumab 150 mg. In this group, the mean SF-36 physical component summary score increased by 12.0 points from baseline to 48.3 at seven days post-dose. SF-36 scores for physical functioning and bodily pain for the canakinumab 150 mg group approached those for the US general population by seven days post-dose and reached norm values by eight weeks post-dose.

**Conclusions:**

Canakinumab 150 mg provided significantly greater and more rapid reduction in pain and signs and symptoms of inflammation compared with triamcinolone acetonide 40 mg. Improvements in HRQoL were seen in both treatment groups with a faster onset with canakinumab 150 mg compared with triamcinolone acetonide 40 mg.

**Trial registration:**

clinicaltrials.gov: NCT00798369.

## Introduction

Gouty arthritis is a common inflammatory arthritis caused by the deposition of monosodium urate (MSU) crystals in joints [1,2]. The deposition and dissolution of the MSU crystals themselves depends on serum urate levels. MSU crystals induce secretion of interleukin-1β (IL-1β), a proinflammatory cytokine that mediates the inflammation that is characteristic of Gouty Arthritis flares and that may remain present between flares [3,4]. There is evidence to suggest that IL-1β may also contribute to joint destruction in Gouty Arthritis [5-7]. Current treatments to control the pain and inflammation associated with acute flares include non-steroidal anti-inflammatory drugs (NSAIDs), colchicine, and corticosteroids [8,9]. However, these treatments are not always effective [10]. In addition, many patients with Gouty Arthritis have underlying comorbidities such as cardiovascular disease, diabetes mellitus, hypertension, liver and renal disease, or gastrointestinal problems, which restrict treatment options or necessitate modifications in the management of Gouty Arthritis [8,11-14]. Furthermore, patients with comorbidities often experience frequent flares. Therefore, there is a need for new therapies to provide effective pain relief in these patients with difficult-to-treat Gouty Arthritis.

Without adequate treatment, Gouty Arthritis can progress into a chronic, deforming, and physically disabling disease through the development of disfiguring tophi, joint destruction, and persistent pain [15,16]. Pain and impaired physical functioning can have a major impact on a patient's health-related quality of life (HRQoL). Results from several observational studies in patients experiencing frequent Gouty Arthritis flares reported HRQoL scores considerably lower than those for men of a similar age in the US general population [17-21]. In addition, patients who experienced more Gouty Arthritis flares and had a greater number of joints involved had a particularly poor HRQoL [18,19]. However, there are no published data regarding the impact of acute flares on HRQoL. The unpredictability of Gouty Arthritis flares further accentuates the impact of this disease on patients. If inappropriately managed, Gouty Arthritis represents a considerable economic burden through lost productivity and the cost of treatment, especially in patients with frequent flares [22,23].

Results of a recent multicenter phase 2 study have shown that canakinumab, a fully human anti-IL-1β monoclonal antibody, can produce rapid reductions in pain in patients with Gouty Arthritis who are unresponsive or intolerant to, or contraindicated for NSAIDs and/or colchicine, and can significantly reduce the risk of recurrent flares [24]. The canakinumab 150 mg dose was found to be superior to triamcinolone acetonide 40 mg on all efficacy measures reported [24]. Here we report further results from this study which confirm the superiority of the 150 mg dose over triamcinolone acetonide including effects on pain assessed using the Likert scale, clinical signs and markers of inflammation (C-reactive protein (CRP) and serum amyloid A protein (SAA)), as well as improvements in HRQoL measures (36-item Short-Form Health Survey (SF-36) and Health Assessment Questionnaire (HAQ)).

## Materials and methods

### Study design

This was an adaptive single-dose, single-blind, active-controlled study. The study was approved by each local independent ethics committee. It was performed in concordance with the ICH Harmonised Tripartite Guidelines for Good Clinical Practice and the ethical principles of the Declaration of Helsinki, and all patients provided written informed consent. Patients were screened at the time of an acute Gouty Arthritis flare; eligible patients were subsequently randomized and received either canakinumab at one of five doses (10, 25, 50, 90, or 150 mg) by subcutaneous (s.c.) injection and saline by intramuscular (i.m.) injection, or i.m. triamcinolone acetonide (40 mg) and a s.c. placebo injection on day 1. Randomization was carried out by means of an interactive voice response system. Patients were not informed of their assigned treatment during the study; wherever possible, treatment was administered by an unblinded pharmacist, nurse or physician who was not involved in any of the study assessments, allowing the investigator to be blinded to treatment. This was the case for 161 of the 200 patients (80.5%) who thus received double-blind treatment.

Patients recorded pain intensity at pre-specified time points (using a Likert scale and visual analog scale (VAS)) and their use of rescue medication during the first seven days of the study. Patients who had difficulty tolerating their pain after the six-hour post-dose pain assessments could take rescue medication consisting of prednisone to a maximum dose of 30 mg once daily for up to five days and acetaminophen 500 mg (up to a maximum of 1 g/dose or 3 g/day) and/or codeine 30 mg (up to a maximum of 30 mg/dose or 180 mg/day) as needed during the first seven days, but not within four hours before a pain assessment. Patients returned to the study center three days (72 hours after study drug administration) seven days, four weeks, and eight weeks post-dose for efficacy and safety assessments. Patients were not informed of their assigned treatment during the study; physicians were not blinded to treatment.

### Patients

Key inclusion criteria were: patients aged 18 to 80 years with a history of at least one previous Gouty Arthritis flare and meeting the American College of Rheumatology 1977 preliminary criteria for the classification of acute arthritis of primary gout; presence of an acute Gouty Arthritis flare for no longer than five days; baseline pain intensity ≥50 mm on the 0 to 100 mm VAS; unresponsive or intolerant to, or contraindicated for NSAIDs and/or colchicine; body mass index (BMI) ≤40 kg/m^2^. Unresponsiveness and intolerance to NSAIDs and/or colchicine and contraindication for NSAIDs and/or colchicine were based on physicians' assessment. Patients on urate-lowering therapy (ULT) were required to be on a stable dose and schedule, with no changes in therapy for four weeks before randomization, and were to be expected to remain on a stable regimen during study participation.

Exclusion criteria included: use of prohibited medications before screening (any ibuprofen in the 4 hours before screening (day 1) or >400 mg in the 8 hours before screening; any acetaminophen in the 4 hours before screening or >1 g in the 24 hours before screening; any aspirin in the 4 hours before screening or >600 mg in the 24 hours before screening; any over-the-counter analgesic aspirin-based or acetaminophen-based combination medication tablets in the 4 hours before screening or >2 tablets in the 24 hours before screening; any diclofenac in the 8 hours before screening or >50 mg in the 24 hours before screening; any naproxen in the 12 hours before screening or >500 mg in the 24 hours before screening; cyclo-oxygenase-2 inhibitors in the 48 hours before screening; other NSAIDs in the 24 hours before screening; systemic corticosteroids in the 24 hours before screening (a dose <10 mg of prednisolone or equivalent was permissible in the 24 hours before screening); intra-articular corticosteroids in the 4 weeks before screening; more than one dose of 0.6 mg colchicine in the 24 hours before screening, if not on a stable dose regimen; anakinra in the 24 hours before screening; rilonacept in the week before screening; other investigational drugs or experimental biologic treatment, other than anakinra or rilonacept, in the 30 days (or 3 months for monoclonal antibodies) or five half-lives before screening, whichever was longer, or as instructed by local regulations; any tumor necrosis factor inhibitor in the 3 months before randomization; rheumatoid, infectious/septic or other inflammatory arthritis; severe renal function impairment; drug allergies; idiopathic thrombocytopenic purpura; contraindication to i.m. injections; donation or loss of ≥400 mL of blood in the 8 weeks before dosing; live vaccination in the 3 months before the start of the study; known presence or suspicion of active or recurrent infection at enrolment; evidence of active pulmonary disease; requirement for administration of antibiotics against latent tuberculosis; risk factors for tuberculosis; any surgical or underlying hepatic, hematological, pulmonary, infectious or gastrointestinal condition that compromised the patient's immune system and/or placed them at unacceptable risk if they received immunomodulatory therapy; long QT syndrome or QTc >450 ms for men and >470 ms for women at screening or baseline; significant medical problems, e.g. uncontrolled hypertension, uncontrolled diabetes, thyroid disease, history of malignancy of any organ system within the preceding 5 years; pregnant or nursing (lactating) women; women who were physiologically capable of becoming pregnant unless they were using an acceptable method of contraception; familial and social conditions rendering regular medical assessment impractical.

### Assessment and definition of response

Study assessments made at baseline and each subsequent scheduled clinic visit (72 hours, 7 days, 4 weeks, and 8 weeks post-dose) included: patient assessment of pain intensity using a 5-point Likert scale (recording no (0), mild (1), moderate (2), severe (3), and extreme (4) pain) and a VAS (ranging from no pain (0 mm) to unbearable pain (100 mm)); the physician's assessment of tenderness, swelling, and erythema in the target joint; and physician and patient global assessments of response to treatment. In addition, blood samples were collected for assessment of blood chemistry (including CRP and SAA levels) and hematology. Adverse events (AEs) were reported throughout the study and physicians assessed local tolerability at sites of s.c. and i.m. injections at each scheduled visit. Blood samples were assessed for anti-canakinumab antibodies at baseline and at eight weeks post-dose using a validated Biacore^® ^binding assay (Biacore International AB, Uppsala, Sweden) [25].

Pain intensity scores (according to the Likert scale and VAS) were recorded by patients in their diaries at 6, 12, 24, and 48 hours, and 4, 5, and 6 days post-dose, and during scheduled clinic visits at 72 hours, 7 days, 4 weeks, and 8 weeks post-dose. Physicians assessed inflammation in the target joint using the following tenderness and swelling scales: tenderness rated as none, 'no pain'; mild, 'pain'; moderate, 'pain and winces'; severe, 'pain; winces and withdraws'; and swelling rated as none, 'no swelling'; mild, 'palpable'; moderate, 'visible'; and severe, 'bulging beyond the joint margins'. Erythema was assessed as 'absent', 'present' or 'not assessable'. Physicians rated response to treatment as 'very good', 'good', 'fair', 'poor', or 'very poor'; and patients rated response to treatment as 'excellent', 'good', 'acceptable', 'slight' or 'poor'. Subsequent flares were identified from patient-reported signs and symptoms of Gouty Arthritis.

### HRQoL instruments

HRQoL was assessed at baseline, and at seven days and eight weeks post-dose using the SF-36 (acute version 2) and the Health Assessment Questionnaire (HAQ). These were exploratory endpoints.

#### 36-item Short-Form Health Survey

SF-36 measures the impact of disease on overall quality of life and consists of eight individual domains that can be grouped to derive a physical component summary (PCS) (composed of physical functioning, role-physical, bodily pain and general health) and a mental component summary (MCS) (composed of vitality, social functioning, role-emotional and mental health) [26]. Scores range from 0 to 100, where 0 represents the worst possible health and 100 is perfect health [27]. This instrument has been validated for use in patients with Gouty Arthritis [28,29]. This study employed the acute (one-week) recall version of SF-36 version 2 [30]. This more recently developed acute form of the 36-item questionnaire was designed for applications in which health status is measured weekly or biweekly. It was created by changing the recall period for six of the eight scales [Role-Physical (RP), Bodily Pain (BP), Vitality (VT), Social Functioning (SF), Role-Emotional (RE) and Mental Health (MH)] from "the past four weeks" to "the past week". The other two scales, Physical Functioning (PF) and General Health (GH), do not have a recall period; the items and instructions for these scales are identical across acute and standard forms. This acute version of SF-36 has been shown to be more sensitive to recent changes in health status. Results were plotted as spidergrams, as recommended by Strand *et al. *[31].

#### Health Assessment Questionnaire

The HAQ assesses a patient's physical ability, functional status, and quality of life through 20 questions concerning difficulty in performing eight common activities of daily living [32,33]. Patients choose from four response categories with scoring of 0 to 3, ranging from 'without any difficulty' (0) to 'unable to do' (3).

### Statistical analysis

In this paper we report the results of secondary efficacy endpoints with respect to reduction of signs and symptoms of inflammation during flares and exploratory endpoints regarding HRQoL. Results for the primary endpoint and for other secondary endpoints have been reported previously and are not included in this paper [24].

Differences in the reduction in pain (Likert scale) were analyzed using an analysis of covariance with treatment group, baseline Likert scale score, and baseline BMI as covariates, while differences in joint tenderness, swelling and erythema were assessed using proportional odds regression with treatment group, baseline physician assessment, and baseline BMI as covariates. Differences in physician and patient global assessments were assessed using proportional odds regression with treatment group and BMI at baseline as covariates. The percentage of patients with no/mild pain was analyzed using a logistical regression model with baseline Likert score and BMI as covariates. The percentage of patients with normalized CRP and SAA concentration values was analyzed using a logistical regression model with baseline CRP/SAA concentration value and BMI as covariates. Mean and standard deviation (SD) were determined for SF-36 PCS, MCS and subscale scores. Descriptive analyses are provided for HAQ scores. All covariates were defined *a priori*. No adjustment was made for multiplicity.

All efficacy end points were analyzed using the full analysis set (i.e. all randomized patients who received study drug had at least one post-baseline VAS assessment) and safety assessments were based on the safety analysis set (i.e. all randomized patients who received study drug and had at least one post-baseline safety assessment).

## Results

**Figure 1:**
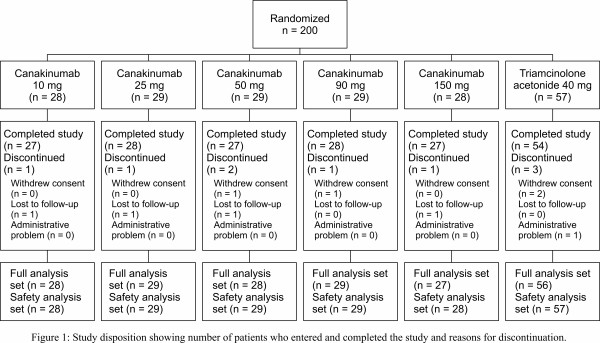
**Study disposition**. Number of patients who entered and completed the study and reasons for discontinuation.

Between November 2008 and May 2009, 200 patients from 89 centers in 11 countries (Argentina, Belgium, Canada, France, Germany, Poland, Russia, Switzerland, Turkey, the UK and the USA) were enrolled and 191 patients completed the study (Figure 1). The demographic and baseline disease characteristics of patients enrolled on this study were generally well balanced across treatment groups and have been described previously [24] (for details see Supplementary table S1 in Additional file 1). Most patients had had multiple acute Gouty Arthritis flares in the preceding 12 months (mean number of flares in each treatment group, 3.9 to 6.8). There was a baseline imbalance in pain intensity between groups, with mean scores being lowest in the canakinumab 150 mg group; the imbalance was significant for VAS score (*P *= 0.005), but not for the Likert scale scores. Physician baseline assessments of joint tenderness, swelling, and erythema were generally well balanced among treatment groups.

### Pain reduction

**Figure 2:**
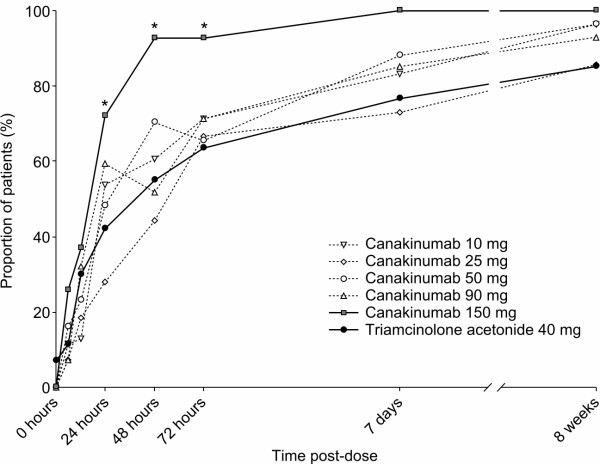
**Percentage of patients experiencing no or mild pain following administration of study medication**. Pain assessments made using a 5-point Likert scale. *P < 0.05 for canakinumab 150 mg vs triamcinolone acetonide 40 mg. CI, confidence interval; LS, least-squares.

At baseline, 98% of patients were suffering from moderate, severe, or extreme pain in the target joint (Likert assessment) (see Supplementary table S1 in Additional file 1). Reductions in the percentage of patients with moderate/severe/extreme pain were seen in all treatment groups from six hours post-dose onwards. The percentage of patients with no or mild pain was numerically greater in most canakinumab groups compared with triamcinolone acetonide from 24 to 72 hours post-dose and the difference was statistically significant for the 150 mg group at these time points (*P *< 0.05) (Figure 2). The reduction in pain intensity from baseline was also significantly greater for canakinumab 150 mg compared with triamcinolone acetonide from 48 hours post-dose to 7 days post-dose (Figure 2).

### Reduction in signs of inflammation

#### Physician and patient assessments

**Figure 3:**
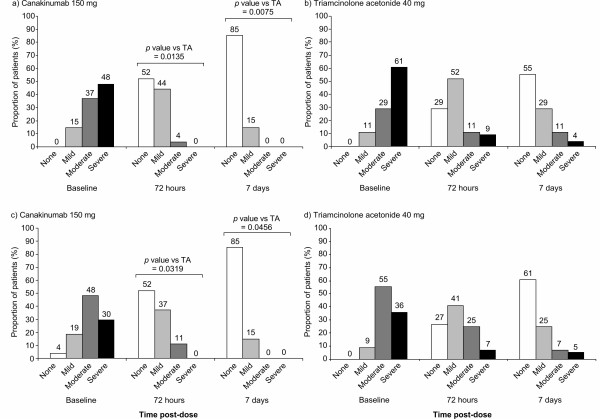
**Reduction in joint inflammation following administration of study medication**. Physician's assessment of joint tenderness in patients receiving canakinumab 150 mg **(a) **or triamcinolone acetonide (TA) 40 mg **(b) **and physician's assessment of joint swelling in patients receiving canakinumab 150 mg **(c) **or triamcinolone acetonide 40 mg **(d)**. Physicians assessed inflammation in the target joint using the following tenderness and swelling scales: tenderness rated as none, 'no pain'; mild, 'pain'; moderate, 'pain and winces'; severe, 'pain; winces and withdraws'; and swelling rated as none, 'no swelling'; mild, 'palpable'; moderate, 'visible'; and severe, 'bulging beyond the joint margins'. Percentages are rounded to one unit therefore numbers at each time point do not necessarily add to 100. TA, triamcinolone acetonide.

**Figure 4:**
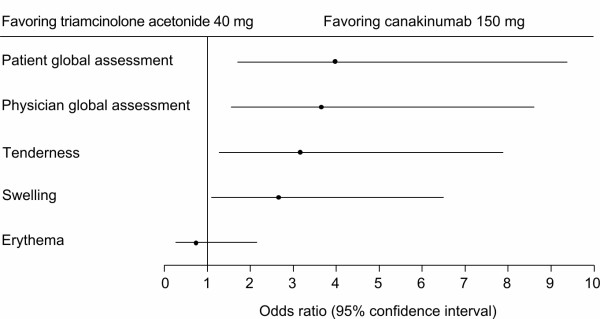
**Physician's and patient's global assessment of response and clinical signs of inflammation (72 hours post-dose)**.

At baseline, most patients had moderate or severe tenderness (85% of patients), moderate or severe joint swelling (85% of patients), and/or erythema (83% of patients). All treatments reduced visible signs of inflammation in the target joint by 72 hours post-dose (the first assessment). At this time point, patients treated with canakinumab 150 mg had a statistically significant lower score on the Likert scale for tenderness and for swelling compared with patients receiving triamcinolone acetonide and the difference between treatments remained statistically significant at seven days post-dose (Figures 3 and 4). Erythema was absent in 74.1% of patients receiving canakinumab 150 mg and 69.6% of patients receiving triamcinolone acetonide at 72 hours post-dose and in 96.3% (canakinumab 150 mg) and 83.9% (triamcinolone acetonide) of patients at seven days post-dose. At 72 hours post-dose canakinumab 150 mg was also associated with statistically significant better responses to treatment according to patient global self-assessment and physician global assessment compared with patients treated with triamcinolone acetonide (Figure 4).

#### Inflammatory markers

At baseline, CRP levels and SAA levels were above the upper limit of the normal range in the majority of patients (CRP, 79.2%; SAA, 64.0%). At seven days post-dose, CRP levels were normalized (≤3.0 mg/L) in 46.4 to 72.4% of patients in the canakinumab groups vs 41.1% in the triamcinolone acetonide group (Table 1). For the canakinumab 150 mg group, the percentage of patients with normalized CRP levels was significantly greater than that in the triamcinolone acetonide group at seven days, four weeks, and eight weeks post-dose (*P *< 0.05). SAA levels were normalized (≤6.7 mg/L) in 63.0 to 75.9% of canakinumab-treated patients compared with 44.6% of patients receiving triamcinolone acetonide at seven days post-dose (Table 1). From this time point onwards the percentage of patients with normalized SAA levels was numerically greater for all canakinumab groups compared with triamcinolone acetonide, but the difference did not reach statistical significance for most doses or time points (Table 1).

**Table 1:** Percentage of patients achieving normalization^1 ^of C-reactive protein levels and serum amyloid A protein levels

Variable	Canakinumab 10 mg *N *= 28	Canakinumab 25 mg *N *= 29	Canakinumab 50 mg *N *= 28	Canakinumab 90 mg *N *= 29	Canakinumab 150 mg *N *= 27	Triamcinolone acetonide 40 mg *N *= 56
CRP						
Baseline	4 (14.3)	8 (27.6)	6 (21.4)	6 (20.7)	5 (18.5)	12 (21.4)
3 days post-dose	6 (22.2)	13 (46.4)	9 (34.6)	9 (32.1)	11 (44.0)	19 (35.8)
7 days post-dose	13 (46.4)	21 (72.4)*	16 (57.1)	19 (67.9)*	19 (70.4)*	23 (41.8)
4 weeks post-dose	21 (77.8)*	22 (78.6)*	20 (74.1)*	18 (64.3)	20 (74.1)*	27 (49.1)
8 weeks post-dose	17 (65.4)*	21 (75.0)*	17 (63.0)	21 (75.0)*	22 (81.5)*	23 (42.6)

SAA						
Baseline	8 (28.6)	9 (32.1)	9 (32.1)	8 (27.6)	9 (33.3)	23 (44.2)
3 days post-dose	15 (57.7)	14 (50.0)	16 (57.1)	13 (46.4)	13 (48.1)	26 (48.1)
7 days post-dose	19 (67.9)	22 (75.9)*	19 (67.9)	21 (75.0)*	17 (65.4)	25 (47.2)
4 weeks post-dose	24 (88.9)*	24 (82.8)	19 (70.4)	20 (71.4)	18 (69.2)	34 (63.0)
8 weeks post-dose	18 (69.2)	21 (80.8)	18 (69.2)	19 (70.4)	20 (74.1)	30 (57.7)

*P < 0.05 vs triamcinolone acetonide 40 mg.

^1^Upper limit of normal: 3 mg/L for CRP, 6.7 mg/L for SAA.

CRP, C-reactive protein; SAA, serum amyloid A protein.

### HRQoL measures

#### 36-item Short-Form Health Survey (acute version 2)

At baseline, mean SF-36 PCS and mean MCS were well below those of the US general population: PCS, 30.0 to 36.1 (US general population, mean ± SD 50.0 ± 10.0) and MCS, 42.9 to 48.2 (US general population, mean ± SD 50.0 ± 10.0) [27]. Similarly mean scores for the individual SF-36 domains were much lower than those for the general US population: physical functioning, 31.1 to 41.5 (US general population, 84.2); role-physical, 31.3 to 53.0 (US general population, 80.9); bodily pain, 23.5 to 36.0 (US general population, 75.2); general health, 53.5 to 65.4 (US general population, 71.9); vitality, 41.3 to 53.9 (US general population, 60.9); social functioning, 47.7 to 62.5 (US general population, 83.3); role-emotional, 54.6 to 66.5 (US general population, 81.3); mental health, 58.1 to 67.9 (US general population, 74.7).

All aspects of physical health improved in all treatment groups over the first seven days post-dose, as reflected in increases in PCS, and were greatest for the canakinumab 150 mg group. In this group, mean (± SD) PCS increased by 12.0 ± 10.0 points from baseline to 48.3 ± 8.6 at seven days post-dose and exceeded that of the US general population by eight weeks post-dose, having a score of 52.8 ± 6.7. A more modest increase of 8.5 ± 10.4 points to 41.9 ± 9.5 at seven days post-dose was reported for the triamcinolone acetonide group and at eight weeks post-dose, the score (47.1 ± 11.2) remained below that of the US general population.

**Figure 5:**
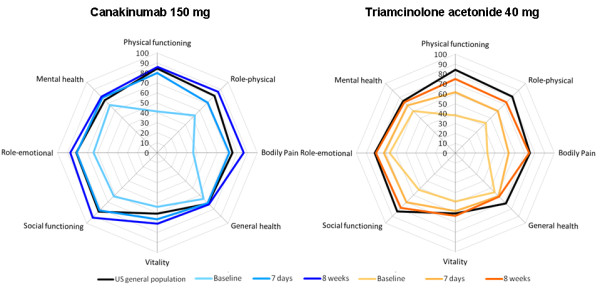
**Spidergrams showing HRQoL improvement (SF-36 scores): canakinumab 150 mg (A); triamcinolone acetonide 40 mg (B)**. Acute version 2 of SF-36, 36-item Short-Form Health Survey; HRQoL, health-related quality of life.

In the canakinumab 150 mg group, the greatest improvements in physical function were seen in the physical functioning and bodily pain domains (Figure 5a). Mean physical functioning scores rapidly improved in the canakinumab 150 mg group from 41.5 at baseline to 80.0 at seven days post-dose (a mean increase of 39.0 points), and exceeded the value for the US general population by eight weeks post-dose (86.1 vs 84.2 for the US general population). Similar improvements were seen in mean bodily pain scores in the canakinumab 150 mg group from 36.0 at baseline to 72.2 at seven days post-dose (a mean increase of 35.6 points) and 86.6 at eight weeks post-dose (vs 75.2 for the US general population). More modest and slower improvements were observed in the triamcinolone acetonide group and scores remained below those of the US general population at eight weeks post-dose (Figure 5b).

Improvements in mental well-being accompanied these changes in physical health. Mean MCS values increased in all treatment groups and exceeded that of the US general population at eight weeks post-dose in all canakinumab groups and approached that of the US general population for the triamcinolone acetonide group (mean ± SD at eight weeks post-dose: canakinumab, 50.6 ± 8.2 to 53.3 ± 7.4; triamcinolone acetonide, 49.1 ± 11.1). Improvements from baseline to eight weeks post-dose were greater in all canakinumab groups compared with triamcinolone acetonide (5.1 ± 10.1 to 9.5 ± 13.2 vs 4.9 ± 15.7). In the canakinumab 150 mg group, improvements were seen in all individual domains, and the greatest improvement was seen for social functioning (Figure 5a). An increase of 18.8 points from baseline to 81.7 was seen at seven days post-dose for the canakinumab 150 mg group, and a further increase to 91.7 was seen at eight weeks post-dose, which exceeded that of the US general population (83.3). Improvements in social functioning were also seen in the triamcinolone acetonide group but the mean score remained below that of the US general population at eight weeks post-dose (78.1 vs 83.3) (Figure 5b).

#### Health Assessment Questionnaire

Mean HAQ scores at baseline were indicative of mild functional disability and were similar for all groups except the canakinumab 150 mg group (mean for all groups except canakinumab 150 mg, 1.03 to 1.28; canakinumab 150 mg, 0.74; *P *= 0.063). Reductions in disability were seen in all canakinumab and triamcinolone acetonide groups, reflected in reductions in HAQ score from baseline of 0.46 to 0.67 at seven days post-dose and 0.52 to 0.85 at eight weeks post-dose.

### Safety and tolerability

As reported previously [24], all treatments were generally well tolerated. There were no deaths and no patients experienced serious AEs related to the study drugs or discontinued the study owing to AEs. The incidence of patients with AEs was similar for canakinumab (59 out of 143 patients, 41.3%) and triamcinolone acetonide (24 out of 57 patients, 42.1%), and all except two of the AEs were mild or moderate in severity. The incidence of infections was low; 7% of all canakinumab-treated patients and 7% of those in the triamcinolone acetonide group. Anti-canakinumab antibodies were detected in two patients in the canakinumab 150 mg group. In one patient, antibodies were detected at baseline, but were absent at the end of the study. In the second patient, antibodies were detected at baseline and at the end of the study. No anti-canakinumab antibodies were detected in the other treatment groups.

## Discussion

Gouty Arthritis causes severe pain and morbidity [34,35]. In our study, 98% of patients reported having moderate-to-extreme pain at baseline and 68% of patients reported having severe or extreme pain; as reported previously, mean VAS scores at baseline were 66 to 78 mm [24]. These data are in broad agreement with other studies reporting on the severity of pain during an acute flare and indicate clearly that the majority of patients experience severe pain [36-39]. For example, two other studies which have assessed pain in patients with acute Gouty Arthritis using a 5-point Likert scale have reported that 49 to 53% [38] and 89% of patients had severe or extreme pain at baseline [36], whereas two studies which have employed a 0 to 100 mm VAS reported baseline scores of 59 to 62 mm [37] and 74 to 78 mm during activity [39]. These scores suggest that pain associated with acute flares is at least as great or greater than that experienced by patients with osteoarthritis (OA) (mean VAS score, 68 mm [40]) or rheumatoid arthritis (RA) (mean VAS scores of 64 to 67 mm [40] and 62 mm [41] have been reported). Rapid effective pain relief is, therefore, a priority for management of acute Gouty Arthritis.

In our study, canakinumab treatment produced rapid and sustained reductions in pain in patients with acute Gouty Arthritis who were unresponsive or intolerant to, or contraindicated for NSAIDs and/or colchicine. Reductions in pain according to Likert scale scores were seen with all canakinumab doses, with 24 to 67% of patients having no or mild pain by 24 hours post-dose (compared with 38% with triamcinolone acetonide), and reductions in pain in the canakinumab 150 mg group were significantly greater than those reported for triamcinolone acetonide from 48 hours to 7 days post-dose. These results paralleled those we have previously reported for our study using the 0 to 100 mm VAS [24]. Pain relief was rapid, with the LS mean reduction in pain at 48 hours post-dose being 2.0 (according to the Likert scale) and 58.0 mm (according to the VAS assessment) in the canakinumab 150 mg group.

Comparison of results across different studies necessarily needs to be cautious given differences in study design and patient population. However, a meaningful comparison can be made with the results reported by Janssens *et al. *who assessed pain relief in a similar patient population using a 0 to 100 mm VAS scale at 12 hour intervals for up to 90 hours following the first dose of treatment [37]. In this double-blind study, patients were randomized to receive prednisolone 35 mg once daily or naproxen 500 mg twice daily. Pain scores at baseline were approximately 15 mm less than in our study, suggesting that pain was slightly more severe in our study. Reductions in pain score from baseline were comparable for prednisolone and naproxen at all time points and were less than those reported in our study for canakinumab doses of 50 mg or greater or for triamcinolone acetonide at the corresponding times. For example, the mean change from baseline was 10 mm for prednisolone and 8 mm for naproxen at zero to six hours [37], and 20 mm for canakinumab 150 mg and 12 mm for triamcinolone acetonide at six hours in our study. Similarly, at 66 to 78 hours the mean change from baseline was 42 mm for both prednisone and naproxen, comparable with the 43 mm change from baseline observed with triamcinolone acetonide 40 mg at 72 hours in our study; a change from baseline of 63 mm was observed at 72 hours for canakinumab 150 mg. The robust pain reductions described here are in contrast to those reported for another inhibitor of IL-1β signaling in development, which has apparently failed to demonstrate significant improvements in pain (relative to a standard regimen of indomethacin) in patients with acute Gouty Arthritis [42].

Triamcinolone acetonide 40 mg was chosen as the comparator in this study based on the experience of the investigators and the fact that in two countries in which the study was performed, the 40 mg im dose is labelled as the initial dose or usual dose and higher doses were not considered to be acceptable to investigators or the health authorities. Furthermore, according to a recent survey, 72% of prescriptions for triamcinolone acetonide in France, Germany and the UK in 2008 to 2009 were for the 40 mg dosage (IMS Disease Analyser: Prescriptions of triamcinolone acetonide in France, and Germany for August 2009 to August 2010 and in the UK for December 2008 to December 2009, personal communication). However, there are no comparative trials to indicate that the 60 mg dose is more effective than the 40 mg dose. The results of our study indicate that triamcinolone acetonide 40 mg is an effective treatment for acute Gouty Arthritis having at least comparable efficacy to that reported for prednisolone and naproxen by Janssens *et al. *[37]. Thus triamcinolone acetonide 40 mg was an appropriate comparator to use in this study.

The results reported here for canakinumab are in agreement with accumulating evidence suggesting that IL-1β, in addition to mediating inflammation, can stimulate pain directly by activating nociceptors (pain receptors) [43] and indirectly by signaling through complex cascades that upregulate and/or activate other pain stimulating molecules [44]. Moreover, IL-1β release in response to injury can contribute to persistent pain by stimulating neural hyperexcitability and increasing sensitivity to pain (hyperalgesia) [44]. Therefore, inhibition of IL-1β with canakinumab may lead directly to a reduction in pain, as well as indirectly through inhibition of inflammation.

The Special Interest Group for gout outcomes at the Outcome Measures in Rheumatology Clinical Trials (OMERACT) recognized the importance of HRQoL measurement in gout and included it as a core domain for clinical trials for chronic Gouty Arthritis [45]. Undoubtedly, the pain associated with acute flares has a severe impact on quality of life, as was evident in our study and has been reported previously [17-21,46]. We herein report the use of the HRQoL instruments SF-36 acute version and HAQ in acute Gouty Arthritis patients. In our study, mean baseline scores for all SF-36 physical domains and for the PCS were considerably lower than those for the US general population (physical functioning, 31 to 42; role-physical, 31 to 53; bodily pain, 24 to 36; PCS, 30 to 36). These scores suggest considerably reduced physical function and are comparable with those expected for men in the US general population aged >75 years, while most of our study population was of working age with an approximate median age of 50 years. Baseline scores for HAQ were indicative of moderate disability, and were in agreement with the reduced physical function evident from SF-36 scores.

The influence of gouty arthritis on patient HRQoL is becoming increasingly recognized but still there are limited data available for HRQoL during an acute flare. Our results are in agreement with a number of recent studies using the SF-36 scale to assess the HRQoL of Gouty Arthritis patients with differing disease severities [18,19,21,28]. Interestingly, the scores reported in our study are similar to those reported in two studies using patients who were intolerant of or refractory to ULT (who would be expected to have frequent flares) [18,21]. For example, Becker *et al. *reported a mean SF-36 physical functioning score of 46.8, a mean role-physical score of 35.0 and PCS value of 34.2 for a population with a mean age of 59 years and experiencing 0.6 flares per month [18], while Strand *et al. *reported reductions of 30 to 32 points for physical function, role-physical and bodily pain SF-36 domains compared with age- and gender-matched controls [21]. Furthermore, in our study, all aspects of mental health were reduced to lower than US norms and were in agreement with scores reported by Strand *et al. *These authors suggested that the HRQoL for their patient population was comparable with that of patients suffering from long-standing RA or active systemic lupus erythematosus, and was much lower than that for patients with OA or cardiac angina [21]. This outcome provides initial insights into the impact of acute Gouty Arthritis flares on HRQoL in an acute setting, which has not been studied so far. Long-term follow up of HRQoL in Gouty Arthritis patients, using other more specialized HRQoL questionnaires, such as the EQ-5D, may yield further insights into the long-term outcomes of the different treatments in the future.

In all groups, treatment was associated with dramatic improvements in HRQoL, particularly relating to physical function, by seven days post-dose. The greatest improvements were seen in the physical functioning and bodily pain domains, and were particularly marked in the canakinumab 150 mg group. By seven days post-dose, mean scores almost reached or were equivalent to those for the US general population for the canakinumab 150 mg group on all domains and reached or exceeded scores for the general population on most domains by eight weeks post-dose. In contrast, in the triamcinolone acetonide group, mean scores remained 10 to 20 points below those of the general population at seven days post-dose and approached those of the general population by eight weeks post-dose. This is the first study to report the impact of anti-inflammatory therapy on quality of life in patients with acute Gouty Arthritis and demonstrates the significant value of potent anti-inflammatory therapy.

We also report that canakinumab achieved rapid reductions in inflammation. Physician-assessed visible signs of inflammation in the joint were reduced by 72 hours post-dose following treatment with canakinumab, and greater reductions in joint tenderness and swelling were observed throughout the study with canakinumab 150 mg compared with triamcinolone acetonide. In addition, treatment with canakinumab 150 mg was associated with a statistically significant better response to treatment according to patient global self-assessment (*P *= 0.002) and physician global assessment (*P *= 0.003) compared with patients treated with triamcinolone acetonide. Previous studies of NSAIDs and corticosteroids have reported the effects of treatment on visible signs of inflammation [36,38,47-50]. However, these studies have used different scales to measure effects and hence results are not comparable with those we report here.

Rapid reductions in visible signs of inflammation were accompanied by significant reductions in levels of the main acute phase inflammatory proteins, CRP and SAA. By seven days post-dose, CRP and SAA levels were normalized in 70% (CRP) and 63% (SAA) of patients receiving canakinumab 150 mg. This supports evidence indicating that IL-1β contributes to the regulation of the production of CRP and SAA [51]. By inhibiting the activity of IL-1β and its generation from pro-IL-1β, canakinumab decreases the production of CRP and SAA. This may be clinically important, not only for its impact on acute Gouty Arthritis and the risk of recurrent flares, but also because of the possible role of elevated CRP and SAA levels and IL-1β in the development of cardiovascular disease [52-54]. As Gouty Arthritis may be an independent risk factor for coronary artery disease [55] and for cardiovascular mortality [56], reducing the levels of these inflammatory proteins may be of additional benefit in patients with Gouty Arthritis.

Our study has a number of limitations. First, the study involved patients who were unresponsive or intolerant to or contraindicated for NSAIDs and/or colchicine, based on their physician's assessment. Physicians made this assessment based on an interview with the patient and from the patient's medical history. We chose to ask physicians to make these judgments so that the study reflects routine clinical practice and would provide an insight into the characteristics of patients for whom physicians consider standard anti-inflammatory therapy to be inappropriate. Second, in this early phase II study the effect on HRQoL was included as an explorative objective. This was limited to assessments made in an acute setting using the acute version 2 of SF-36 and the HAQ. Further studies including HRQoL assessments targeting longer-term outcomes and in specific patient populations will be required in the future. Third, the study was open to patients who were within five days of the onset of their flare. Thus by 72 hours post-dose, it could be expected that the flare would have started to resolve independently of treatment in some patients. However, the proportion of patients for whom this could apply was very small as the majority of patients (81%) entered the study within three days of onset of flare. Furthermore, the proportion of patients entering the study within four or five days of onset of flare was similar across treatment groups. Thus the differences between canakinumab 150 mg and triamcinolone acetonide observed in this study reflect differences in the efficacy of the two treatments.

## Conclusions

Acute Gouty Arthritis flares are often associated with excruciating pain that can be associated with disability and reduced physical functioning. The results of this study indicate that anti-inflammatory therapy with canakinumab 150 mg produces rapid pain relief, relieves signs and symptoms of inflammation and results in rapid clinically significant improvements in quality of life, especially relating to physical function (as assessed using the SF-36 acute version 2). These results are particularly important for patients with acute Gouty Arthritis who are unable to achieve adequate responses to standard therapies, or to tolerate standard therapies. The canakinumab 150 mg dose is being investigated further in ongoing phase III clinical trials.

## Abbreviations

AE: adverse event; BMI: body mass index; CI: confidence interval; CRP: C-reactive protein; HAQ: Health Assessment Questionnaire; HRQoL: health-related quality of life; IL-1β: interleukin-1β; LS: least-squares; MCS: mental component summary; MSU: monosodium urate; NSAID: non-steroidal anti-inflammatory drug; OA: osteoarthritis; OMERACT: Outcome Measures in Rheumatology Clinical Trials; PCS: physical component summary; RA: rheumatoid arthritis; SAA: serum amyloid A protein; SF-36: 36-item Short-Form Health Survey; ULN: upper limit of the normal range; ULT: urate-lowering therapy; VAS: visual analog scale.

## Competing interests

Dr Schlesinger reports having received lecture fees from Takeda and grant support from the UMDNJ foundation and Novartis Pharmaceuticals as well as fees for serving on advisory boards: Novartis, Takeda, Savient, URL Pharma and EnzymeRx. Dr Pikhlak reports having received consulting fees from Novartis. Drs Sallstig, Richard, and Arulmani and Ms Murphy are employees of Novartis and report having equity interests in Novartis. Dr So reports having received consulting fees from Novartis, Wyeth, and Roche, having equity interests in Pfizer and having received lecture fees from Bristol Myers Squibb, and grant support from Fonds National Suisse. Drs De Meulemeester and Yücel declare that they have no competing interests.

## Authors' contributions

The study was designed by AS, NS, PS, DR, VM and UA, and data were gathered and analyzed by Novartis. All authors vouch for the accuracy of the data and the analysis, and contributed to the interpretation of the data and were involved in the decision to publish. All authors were involved in discussing the content of the manuscript and deciding which data and interpretations were to be included. All authors approved the submitted paper. The contribution of all those who do not meet the criteria for authorship are acknowledged in the paper.

## Supplementary Material

Additional file 1 **Supplementary Table S1: Demographic and baseline characteristics**. Table giving demographic and baseline characteristics of the study population by treatment group. click here for file
