# Supplementary material for: Canakinumab relieves symptoms of acute flares and improves health-related quality of life in patients with difficult-to-treat Gouty Arthritis by suppressing inflammation: results of a randomized, dose-ranging study
Source: Arthritis Res Ther. 2011 Mar 25;13(2):R53. doi: 10.1186/ar3297 (PMC3132043; doi:10.1186/ar3297)
Supplement: Additional file 1 — Supplementary Table S1: Demographic and baseline characteristics. Table giving demographic and baseline characteristics of the study population by treatment group. [file ar3297-S1.DOC]

**ADDITIONAL FILES**

**File name: Additional file 1**

**File format: word**

**Title:** Additional Table: Demographic and baseline characteristics.

**Description:** Table giving demographic and baseline characteristics of the study population by treatment group.

| Variable | Canakinumab 10 mg  N = 28 | Canakinumab 25 mg  N = 29 | Canakinumab 50 mg  N = 29 | Canakinumab 90 mg  N = 29 | Canakinumab 150 mg  N = 28 | Triamcinolone acetonide 40 mg  N = 57 |
| --- | --- | --- | --- | --- | --- | --- |
| Male, n (%) | 26 (92.9) | 26 (89.7) | 27 (93.1) | 24 (82.8) | 28 (100.0) | 55 (96.5) |
| Age, years |  |  |  |  |  |  |
| Mean (SD) | 49.9 (11.12) | 50.5 (10.89) | 54.9 (10.75) | 52.2 (12.38) | 50.6 (15.38) | 52.4 (11.55) |
| Median (range) | 45.5 (34–78) | 49.0 (32–76) | 55.0 (35–76) | 52.0 (28–76) | 51.5 (20–75) | 51.0 (20–76) |
| Age groups, n (%) |  |  |  |  |  |  |
| ≥18–40 years | 6 (21.4) | 4 (13.8) | 3 (10.3) | 5 (17.2) | 8 (28.6) | 7 (12.3) |
| ≥41–64 years | 21 (75.0) | 21 (72.4) | 19 (65.5) | 20 (69.0) | 15 (53.6) | 39 (68.4) |
| ≥65–74 years | 0 | 3 (10.3) | 6 (20.7) | 2 (6.9) | 3 (10.7) | 10 (17.5) |
| ≥75 years | 1 (3.6) | 1 (3.4) | 1 (3.4) | 2 (6.9) | 2 (7.1) | 1 (1.8) |
| Race, n (%) |  |  |  |  |  |  |
| Caucasian | 27 (96.4) | 27 (93.1) | 23 (79.3) | 25 (86.2) | 24 (85.7) | 54 (94.7) |
| Black | 1 (3.6) | 1 (3.4) | 3 (10.3) | 4 (13.8) | 1 (3.6) | 3 (5.3) |
| Asian | 0 | 1 (3.4) | 0 | 0 | 2 (7.1) | 0 |
| Other | 0 | 0 | 3 (10.3) | 0 | 1 (3.6) | 0 |
| BMI, kg/m^2^ |  |  |  |  |  |  |
| Mean (SD) | 32.13 (4.81) | 30.55 (4.87) | 32.02 (4.11) | 31.28 (5.59) | 30.24 (4.48) | 30.91 (4.13) |
| Median (range) | 32.58 (23.1–39.8) | 29.35 (23.1–39.2) | 31.54 (21.1–38.8) | 30.64 (23.6–39.6) | 30.81 (20.1–39.0) | 31.12 (20.9–39.5) |
| Classification of gout, n (%) |  |  |  |  |  |  |
| Acute monoarticular gout | 16 (57.1) | 15 (51.7) | 18 (62.1) | 17 (58.6) | 19 (67.9) | 41 (71.9) |
| Acute oligoarticular gout | 9 (32.1) | 10 (34.5) | 9 (31.0) | 9 (31.0) | 6 (21.4) | 13 (22.8) |
| Acute polyarticular gout | 3 (10.7) | 4 (13.8) | 2 (6.9) | 3 (10.3) | 3 (10.7) | 3 (5.3) |
| Number of joints affected by acute attack of gout within the past 5 days, n (%) |  |  |  |  |  |  |
| 1 | 16 (57.1) | 17 (58.6) | 19 (65.5) | 17 (58.6) | 22 (78.6) | 43 (75.4) |
| 2 | 7 (25.0) | 2 (6.9) | 4 (13.8) | 6 (20.7) | 5 (17.9) | 9 (15.8) |
| 3 | 2 (7.1) | 4 (13.8) | 3 (10.3) | 2 (6.9) | 0 | 2 (3.5) |
| 4 | 1 (3.6) | 3 (10.3) | 2 (6.9) | 1 (3.4) | 0 | 1 (1.8) |
| >4 | 2 (7.1) | 3 (10.3) | 1 (3.4) | 3 (10.3) | 1 (3.6) | 2 (3.5) |
| Number of flares in previous year |  |  |  |  |  |  |
| Mean (SD) | 6.8 (8.14) | 6.3 (5.20) | 3.9 (2.59) | 5.9 (4.93) | 5.3 (4.96) | 6.5 (9.88) |
| Median (range) | 4.0 (1–36) | 5.0 (1–24) | 4.0 (0–10) | 5.0 (1–24) | 3.5 (1–24) | 3.0 (0–50) |
| VAS score of pain intensity*^a^ |  |  |  |  |  |  |
| Mean (SD) | 73.8 (11.50) | 77.6 (12.90) | 76.3 (13.38) | 72.6 (10.62) | 65.5 (9.14) | 72.9 (12.78) |
| Median (range) | 73.0 (51–94) | 76.0 (50–98) | 77.0 (50–99) | 74.0 (54–94) | 68.0 (51–81) | 71.0 (52–100) |
| Likert assessment of pain, n (%) |  |  |  |  |  |  |
| None | 0 | 0 | 0 | 0 | 0 | 0 |
| Mild | 0 | 0 | 0 | 0 | 0 | 4 (7.0) |
| Moderate | 8 (28.6) | 6 (20.7) | 8 (27.6) | 7 (24.1) | 14 (50.0) | 15 (26.3) |
| Severe | 19 (67.9) | 18 (62.1) | 17 (58.6) | 18 (62.1) | 14 (50.0) | 32 (56.1) |
| Extreme | 1 (3.6) | 5 (17.2) | 4 (13.8) | 4 (13.8) | 0 | 6 (10.5) |
| Joint tenderness^g^ n (%) |  |  |  |  |  |  |
| None | 0 | 0 | 0 | 0 | 0 | 0 |
| Mild | 1 (3.6) | 5 (17.2) | 8 (27.6) | 7 (24.1) | 4 (14.3) | 6 (10.5) |
| Moderate | 9 (32.1) | 8 (27.6) | 8 (27.6) | 13 (44.8) | 10 (35.7) | 17 (29.8) |
| Severe | 18 (64.3) | 16 (55.2) | 13 (44.8) | 9 (31.0) | 14 (50.0) | 34 (59.6) |
| Joint swelling^h^ n (%) |  |  |  |  |  |  |
| None | 1 (3.6) | 2 (6.9) | 0 | 1 (3.4) | 1 (3.6) | 0 |
| Mild | 2 (7.1) | 2 (6.9) | 6 (20.7) | 4 (13.8) | 5 (17.9) | 6 (10.5) |
| Moderate | 10 (35.7) | 15 (51.7) | 9 (31.0) | 17 (58.6) | 13 (46.4) | 31 (54.4) |
| Severe | 15 (53.6) | 10 (34.5) | 14 (48.3) | 7 (24.1) | 9 (32.1) | 20 (35.1) |
| Erythema n (%) |  |  |  |  |  |  |
| Absent | 5 (17.9) | 1 (3.4) | 8 (27.6) | 7 (24.1) | 3 (10.7) | 8 (14.0) |
| Present | 23 (82.1) | 27 (93.1) | 21 (72.4) | 22 (75.9) | 25 (89.3) | 48 (84.2) |
| Not assessable | 0 | 1 (3.4) | 0 | 0 | 0 | 1 (1.8) |
| SF-36 physical component score |  |  |  |  |  |  |
| Mean (SD) | 30.0 (9.42)^b^ | 31.5 (8.97) | 31.7 (7.99) | 32.4 (9.97) | 36.1 (8.23) | 33.5 (9.17)^b^ |
| Median (range) | 27.4 (15–50) | 30.4 (20–56) | 32.5 (16–48) | 32.9 (17–56) | 34.4 (25–56) | 31.5 (11–60) |
| SF-36 mental component score |  |  |  |  |  |  |
| Mean (SD) | 44.5 (12.84)^b^ | 42.9 (13.97) | 48.2 (15.72) | 46.9 (11.33) | 47.1 (13.50) | 44.7 (15.07)^b^ |
| Median (range) | 41.0 (23–64) | 44.0 (15–64) | 51.4 (15–69) | 50.1 (20–60) | 46.6 (20–68) | 47.3 (9–68) |
| HAQ SDI score |  |  |  |  |  |  |
| Mean (SD) | 1.15 (0.70) | 1.28 (0.66) | 1.03 (0.62) | 1.15 (0.71) | 0.74 (0.68) | 1.09 (0.65)^b^ |
| Median (range) | 1.19 (0–2.8) | 1.38 (0–2.5) | 1.13 (0–2.5) | 1.00 (0–2.4) | 0.69 (0–2.4) | 1.0 (0–2.5) |
| Patients unresponsive or intolerant to NSAIDS and/or colchicine, n (%) | 27 (96.4) | 27 (93.1) | 26 (89.7) | 20 (69.0) | 24 (85.7) | 50 (87.7) |
| Patients for whom NSAIDs and/or colchicine are contraindicated, n (%) | 4 (14.3) | 5 (17.2) | 7 (24.1) | 12 (41.4) | 4 (14.3) | 13 (22.8) |
| CRP, mg/L |  |  |  |  |  |  |
| Mean (SD) | 23.25 (33.324) | 25.07 (35.010) | 32.84 (54.749)^b^ | 29.35 (33.424) | 21.84 (34.146)^b^ | 18.58 (22.925)^b^ |
| Median (range) | 13.70 (1.2–171.0) | 13.70 (0.0–147.0) | 14.00 (0.7–273.0) | 17.30 (0.3–127.0) | 13.20 (1.0–182.0) | 10.90 (0.0–91.7) |
| SAA, mg/L |  |  |  |  |  |  |
| Mean (SD) | 57.65 (157.059) | 94.07 (170.713)^b^ | 101.24 (205.033)^b^ | 82.21 (132.499) | 65.18 (109.273)^b^ | 43.35 (76.510)^d^ |
| Median (range) | 14.25 (1.2–820.0) | 15.50 (1.8–699.0) | 14.40 (2.6–882.0) | 14.70 (1.3–536.0) | 35.10 (2.0–543.0) | 7.85 (1.3–413.0) |
| Creatinine, μmol/L |  |  |  |  |  |  |
| Mean (SD) | 93.7 (19.6) | 90.3 (16.4) | 100.0 (23.7) | 90.2 (21.2) | 93.6 (23.2) | 93.7 (17.9) |
| Median (range) | 94.0 (57–137) | 90.0 (62–135) | 93.0 (72–178) | 88.0 (63–171) | 88.0 (67–164) | 90.0 (55–149) |
| Creatinine clearance^e^ |  |  |  |  |  |  |
| Low | 0 | 1 (3.4) | 1 (3.4) | 0 | 1 (3.6) | 1 (1.8) |
| Normal | 12 (42.9) | 10 (34.5) | 11 (37.9) | 13 (44.8) | 12 (42.9) | 26 (45.6) |
| High | 16 (57.1) | 17 (58.6) | 16 (55.2) | 16 (55.2) | 14 (50.0) | 26 (45.6) |
| Missing | 0 | 1 (3.4) | 1 (3.4) | 0 | 1 (3.6) | 4 (7.0) |
| Estimated glomerular filtration rate, n (%)^f^ |  |  |  |  |  |  |
| >90 mL/min/1.73 m^2^ | 10 (35.7) | 10 (34.5) | 4 (13.8) | 11 (37.9) | 10 (35.7) | 16 (28.1) |
| 60–89 mL/min/1.73 m^2^ | 15 (53.6) | 16 (55.2) | 18 (62.1) | 15 (51.7) | 15 (53.6) | 28 (49.1) |
| 30–59 mL/min/1.73 m^2^ | 3 (10.7) | 2 (6.9) | 6 (20.7) | 3 (10.3) | 2 (7.1) | 9 (15.8) |
| Missing | 0 | 1 (3.4) | 1 (3.4) | 0 | 1 (3.6) | 4 (7.0) |
| Serum urate, mg/dL |  |  |  |  |  |  |
| Mean (SD) | 8.51 (1.71) | 7.75 (1.97)^b^ | 7.67 (2.10)^b^ | 7.53 (2.23) | 7.89 (1.57)^b^ | 7.83 (2.14)^c^ |
| Median (range) | 8.50 (4.99–12.99) | 7.75 (3.90–11.69) | 7.95 (3.90–13.09) | 7.90 (3.29–12.89) | 7.80 (4.50–10.79) | 8.05 (2.40–12.89) |

^*^*P*= 0.005 (analysis of variance F-test).

^a^VAS assessment of pain ranging from no pain (0 mm) to unbearable pain (100 mm).

^b^Data missing for one patient.

^c^Data missing for three patients.

^d^Data missing for five patients.

^e^Calculated using the Cockcroft–Gault formula.

^f^Calculated using the Modification of Diet in Renal Disease formula.

^g^Tenderness was rated as: none, ‘no pain’; mild, ‘pain’; moderate, ‘pain and winces’; or severe, ‘pain, winces and withdraws’.

^h^Swelling was rated as: none, ‘no swelling’; mild, ‘palpable’; moderate, ‘visible’; or severe, ‘bulging beyond the joint margins’.

BMI, body mass index; CRP, C-reactive protein; HAQ SDI, Health Assessment Questionnaire standard disability index; NSAID, non-steroidal anti-inflammatory drug;; SAA, serum amyloid A; SD, standard deviation; SF-36; 36-item Short-Form Health Survey; VAS, visual analog scale.
